# Foundation Models for Remote Sensing Semantic Segmentation: A Review of Architectures, Adaptations, and Prospects

**DOI:** 10.3390/s26154671

**Published:** 2026-07-23

**Authors:** Ming Deng, Yongyi Chen, Guanghai Ding, Shuiwang Li, Xiaolan Xie

**Affiliations:** College of Computer Science and Engineering, Guilin University of Technology, Guilin 541006, China; gutming@glut.edu.cn (M.D.); 13692260076@163.com (Y.C.); dingguanghai2025@163.com (G.D.)

**Keywords:** remote sensing image segmentation, foundation models, parameter-efficient fine-tuning, domain adaptation, self-supervised learning

## Abstract

**Highlights:**

**What are the main findings?**

- Review of four emerging foundation model paradigms for remote sensing image segmentation—Transformer-based architectures, state space models (Mamba), prompt-driven segmentation (SAM), and self-supervised or multimodal pre-training analyzing trade-offs in global context modeling, computational efficiency, and cross-modal representation.
- Synthesis of downstream adaptation strategies, including parameter-efficient fine-tuning (LoRA, adapters), prompt engineering, few-shot and zero-shot learning, open-vocabulary segmentation, and domain adaptation, revealing how each strategy addresses the gap between pre-training and remote sensing requirements.

**What are the implications of the main findings?**

- Identification of fundamental bottlenecks limiting current models, including the tension between representation generality and remote sensing-specific adaptation, multimodal sensor heterogeneity, and insufficiencies in existing evaluation ecosystems and annotation paradigms.
- A forward-looking research roadmap toward remote-sensing-native pre-training, lightweight edge-deployable architectures, and unified open-world geospatial foundation models, providing guidance for future research and practical deployment.

**Abstract:**

Remote sensing image segmentation is a foundational task in Earth observation. With the rapid growth of remote sensing datasets in terms of scale, modality diversity, semantic openness, and spatio-temporal complexity, the field is evolving from task-specific supervised learning toward foundation-model paradigms. Recent advances in foundation models—including Transformer-based architectures, Mamba-based state space models (SSMs), prompt-driven frameworks such as the Segment Anything Model (SAM), and self-supervised or multimodal pre-training—have profoundly reshaped the technical landscape of remote sensing image segmentation. This paper reviews recent progress from the perspectives of dataset evolution, model architectures, and downstream adaptation strategies, covering parameter-efficient fine-tuning, prompt engineering, few-shot and zero-shot learning, open-vocabulary segmentation, and domain adaptation. We further analyze core challenges including the tension between representation generality and remote sensing-specific adaptation, multimodal sensor heterogeneity, and the insufficiency of existing evaluation ecosystems. Finally, we discuss future directions toward remote-sensing-native pre-training, lightweight edge deployment, and unified open-world geospatial foundation models.

## 1. Introduction

As a core task in the field of Earth observation, remote sensing image segmentation aims to achieve pixel-level semantic understanding of imagery acquired from satellite, aerial, and unmanned aerial vehicle (UAV) platforms. It serves as a fundamental technology for applications such as land-cover mapping, urban planning, disaster monitoring, and agricultural estimation. With the rapid increase in the number of Earth observation satellites and the continuous improvement of sensor resolution, remote sensing imagery has experienced explosive growth in spatial scale, spectral dimension, modality diversity, and temporal frequency. Meanwhile, segmentation tasks have evolved from conventional closed-set semantic segmentation toward more complex scenarios involving multimodal understanding, open-vocabulary recognition, change detection, and vision–language interaction. As data modalities and downstream tasks continue to diversify, remote sensing segmentation models are increasingly required to handle stronger semantic variability and cross-domain generalization [1].

Traditional segmentation approaches based on handcrafted features and shallow machine learning models have become increasingly limited when dealing with complex land-cover categories, cluttered backgrounds, and multi-scale targets in remote sensing scenes. Although deep learning methods represented by Convolutional Neural Networks (CNNs) have achieved significant advances in receptive field expansion and hierarchical feature modeling, their inherent local perception bias makes it difficult to capture the cross-regional long-range spatial dependencies present in remote sensing imagery [2]. More importantly, conventional task-specific supervised paradigms increasingly struggle to generalize across heterogeneous sensors, geographic regions, and downstream tasks under limited annotation conditions.

The emergence of foundation model architectures, such as Vision Transformer (ViT) [3], Segment Anything Model (SAM) [4], and Multimodal Large Language Models (MLLMs) [5], has fundamentally changed how visual knowledge is learned, generalized, and transferred across tasks. Through large-scale pre-training, these models provide stronger cross-task transferability and semantic generalization for remote sensing image segmentation [6]. Meanwhile, the rapid evolution of remote sensing datasets toward larger scales, richer modalities, and more open semantic spaces has further accelerated the transition from task-specific supervised models toward foundation-model paradigms. However, directly transferring general foundation models to remote sensing scenarios remains challenging due to the strong heterogeneity and domain discrepancy of remote sensing imagery.

**Figure 1:**
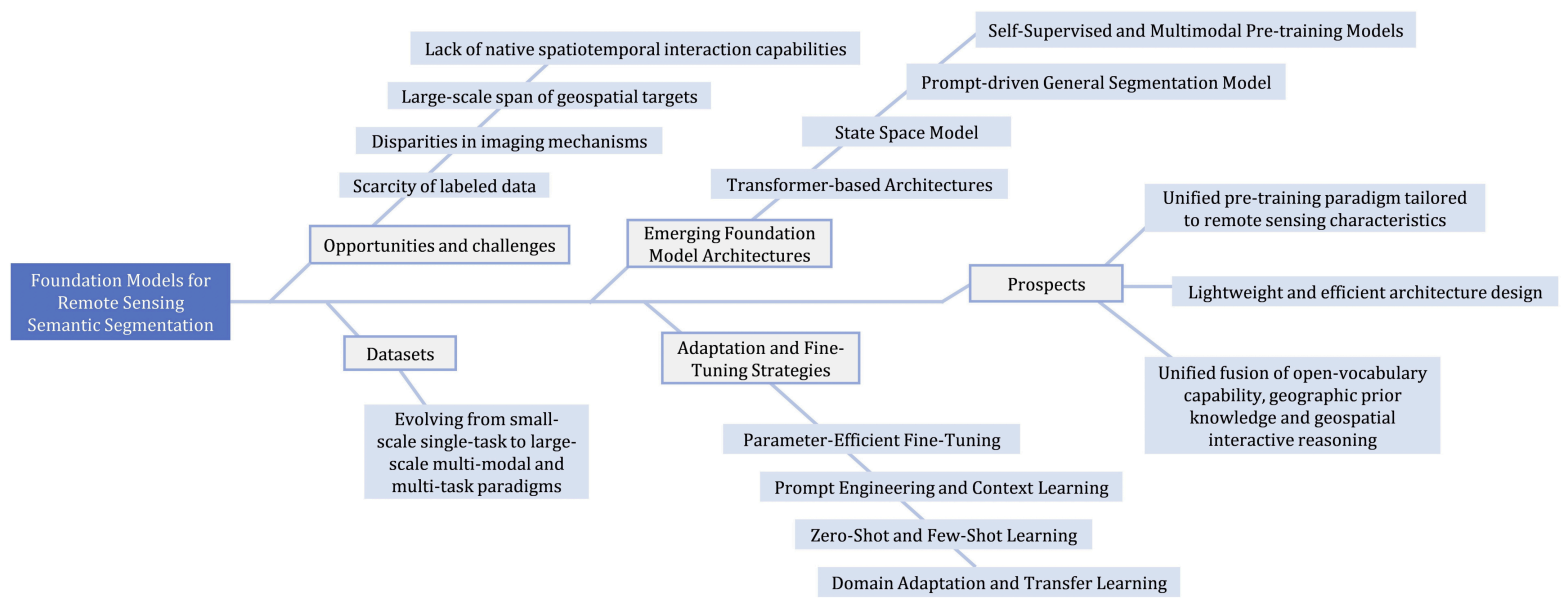
Overall framework of this review on foundation models for remote sensing semantic segmentation, covering architectures, adaptation strategies, and future prospects.

To provide an overview of this rapidly evolving field, Figure 1 presents a unified research landscape and technical roadmap. Table 1 summarizes representative foundation-model paradigms and explicitly distinguishes their roles in the remote sensing segmentation pipeline.

Despite their strong generalization capability, remote sensing imagery differs fundamentally from natural images in imaging mechanisms, modality heterogeneity, and spatial structure. These discrepancies introduce substantial gaps between general-purpose pre-training objectives and downstream remote sensing requirements. Consequently, a central challenge in current research lies not only in improving cross-domain generalization, but also in achieving robust and efficient adaptation under heterogeneous remote sensing conditions. Recent advances in large-scale pre-training and efficient adaptation strategies have further accelerated the development of foundation models for remote sensing image segmentation, enabling improved transferability and scalability across diverse remote sensing scenarios.

In recent years, several related surveys have summarized the rapidly developing field of remote sensing foundation models and semantic segmentation from different perspectives. These surveys can be broadly grouped into two categories: some provide broad overviews of foundation models for remote sensing and Earth observation [12], while others review deep-learning-based semantic segmentation methods for remote sensing images [13]. In contrast, this review focuses on the intersection of these two lines of research, namely foundation models for remote sensing semantic segmentation. By connecting dataset evolution, foundation-model architectures, and downstream adaptation strategies, this review aims to clarify how foundation-model paradigms reshape remote sensing segmentation.

This review summarizes recent research progress on foundation models for remote sensing image segmentation, with the main contributions outlined as follows:

1. This review connects dataset evolution with the emergence of foundation-model paradigms, emphasizing how increasing scale, modality diversity, and semantic openness raise new requirements for generalizable remote sensing segmentation models.
2. It compares representative foundation-model architectures, including Transformer-based models, hybrid CNN–Transformer frameworks, state space models, prompt-driven segmentation models, and self-supervised or multimodal pre-training paradigms.
3. It analyzes downstream adaptation strategies under heterogeneous remote sensing conditions, including parameter-efficient fine-tuning, prompt engineering, few-shot learning, open-vocabulary segmentation, and domain adaptation, with emphasis on the remaining gap between general-purpose pre-training and remote sensing-specific requirements.

## 2. Datasets for Remote Sensing Image Segmentation

The development of remote sensing image segmentation datasets reflects the continuous expansion of task complexity and suggests an increasing demand for more generalizable model paradigms. Early representative datasets, such as ISPRS Vaihingen/Potsdam (2012) [14] and Massachusetts Roads/Buildings (2013) [15], typically feature limited data scales, relatively fixed sensor settings, and localized geographic coverage, with tasks mainly focused on standard semantic segmentation. Under these relatively constrained benchmark conditions, dataset-specific supervised models can often achieve strong performance, but such performance is largely tied to fixed categories, sensors, and regions rather than broad cross-domain generalization.

The intermediate stage further expanded the benchmark landscape. Datasets and benchmarks including the SpaceNet challenge series [16], DeepGlobe (2018) [17], iSAID (2019) [18], and UAVid (2020) [19] reflect this expansion from different perspectives. These developments cover large-scale satellite benchmarks, broader land-cover segmentation and practical application scenarios, instance-wise labeling, and urban scene parsing with UAV imagery. Collectively, these datasets demonstrate that remote sensing segmentation research has gradually moved beyond limited small-scale urban benchmarks toward more diverse, application-driven research scenarios.

In recent years, dataset evolution has become more pronounced along three dimensions. First, data scale has increased substantially, as represented by large-scale pixel-level datasets such as Five-Billion-Pixels (2023) [20]. Second, benchmark tasks have become increasingly application-oriented, extending segmentation evaluation to disaster and environmental monitoring, as illustrated by WorldFloods (2023) [21]. Third, recent datasets have increasingly incorporated multimodal supervision, particularly language-guided or instruction-driven segmentation settings. For example, GeoSeg-1M [22] extends remote sensing segmentation toward instruction-driven and unified open-world geospatial segmentation, while language-guided referring segmentation datasets such as RefSegRS [23] and NWPU-Refer [24] further reflect the growing role of vision–language supervision in this field. Based on these observations, the evolution of dataset complexity can be understood not as a simple linear increase in data size, but as a co-evolution of dataset scale, task diversity, and modality richness.

As benchmarks cover larger areas, more diverse scenes, and richer forms of supervision, models are increasingly expected to work beyond the specific regions, sensors, or label spaces on which they are trained. In this context, purely task-specific models trained from scratch may become less effective when transferred to heterogeneous sensors, diverse geographic regions, or cross-task scenarios. This shift provides an important motivation for introducing foundation-model paradigms into remote sensing image segmentation.

**Figure 2:**
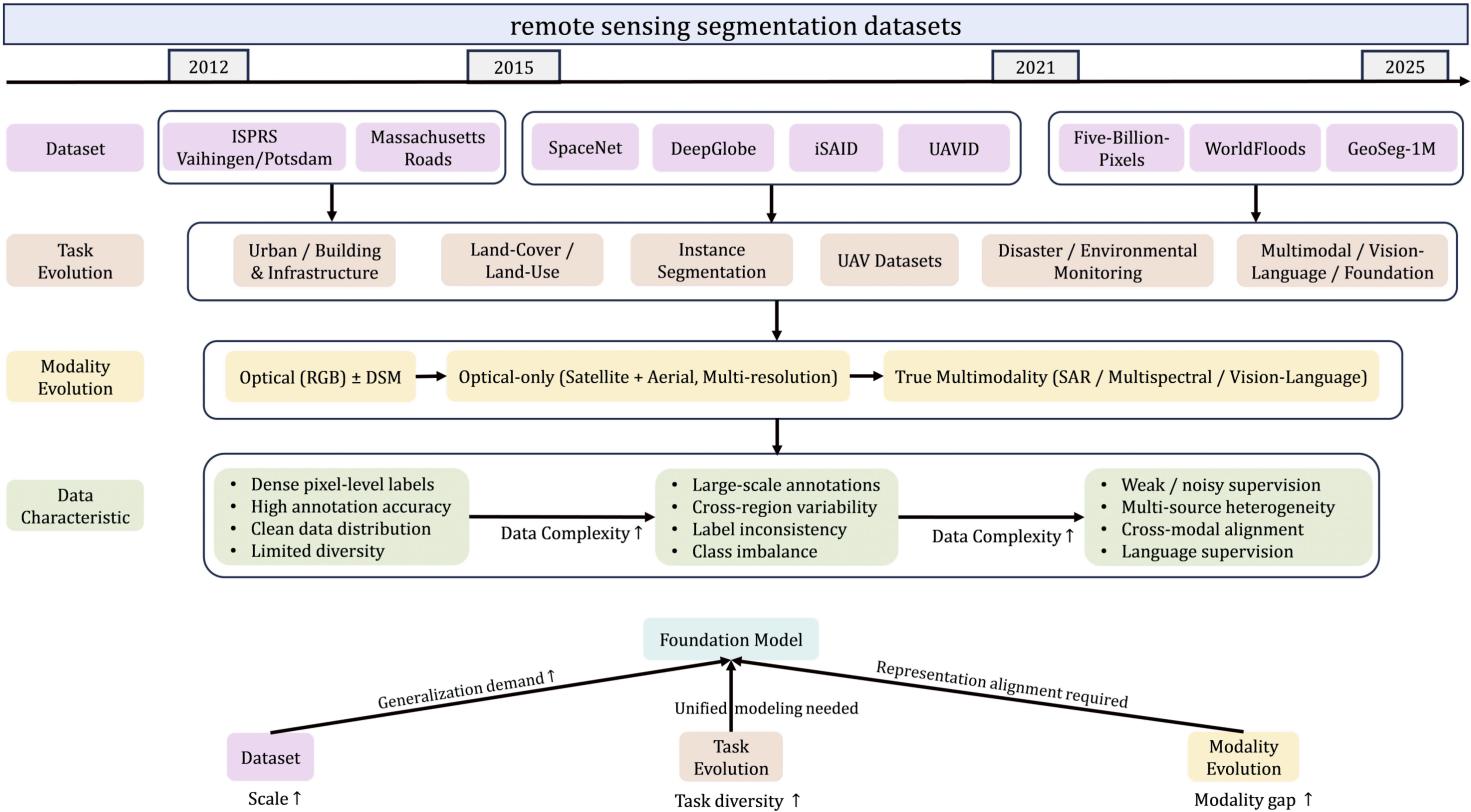
Evolution of remote sensing datasets and their implications for foundation model development. Upward arrows indicate increasing trends.

The above trends are summarized in Figure 2, which shows the joint evolution of dataset scale, task diversity, modality composition, and data characteristics, together with their implications for generalization, unified modeling, and cross-modal representation alignment. Representative datasets are further summarized in Table 2. The datasets are grouped by their primary task orientation and application context, covering urban/building and infrastructure mapping, instance segmentation, land-cover and land-use mapping, disaster and environmental monitoring, UAV-based datasets, and recent multimodal, vision–language, or foundation-model-oriented datasets. These categories are not strictly mutually exclusive, as some datasets are commonly used in multiple segmentation settings. Beyond task type and data scale, these datasets also differ substantially in geographic coverage, sensor source, and spatial resolution. Such differences affect model transferability because aerial, UAV, satellite, synthetic aperture radar (SAR), multispectral, and optical imagery may exhibit different spatial details, band characteristics, noise patterns, and imaging mechanisms. Spatial resolution further influences the apparent size, texture, and boundary clarity of ground objects. Therefore, cross-region, cross-resolution, and cross-sensor differences should be considered when interpreting benchmark results and assessing the practical generalization ability of foundation models. Based on this dataset evolution, Section 3 reviews current foundation model architectures for remote sensing semantic segmentation.

## 3. Emerging Foundation Model Architectures

**Figure 3:**
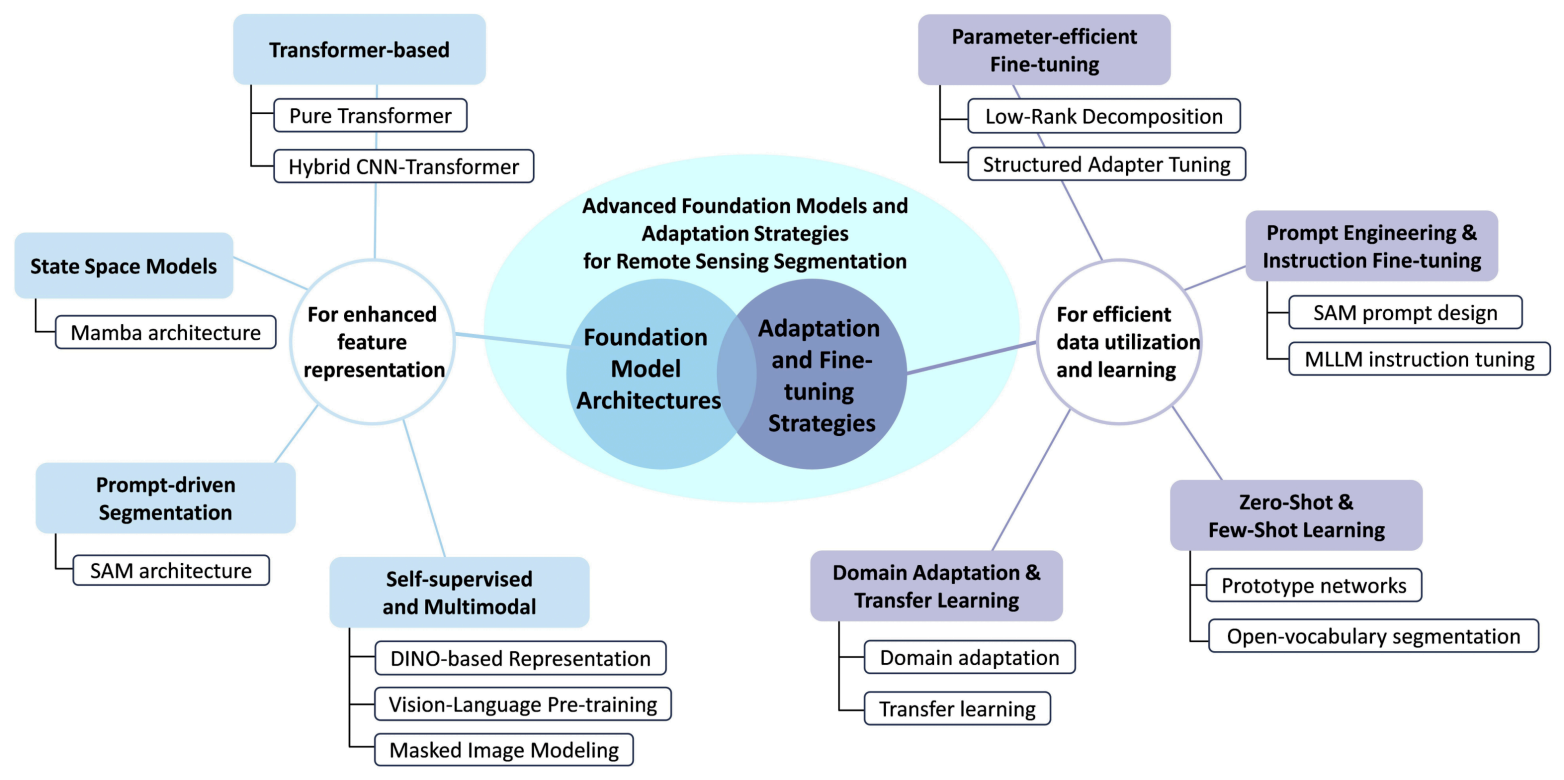
Overview of foundation model architectures and adaptation strategies for remote sensing image segmentation.

To provide a unified conceptual framework, Figure 3 illustrates the overall landscape of foundation models for remote sensing image segmentation, bridging both architectural design (Section 3) and adaptation strategies (Section 4).

Specifically, this figure organizes the field along two tightly coupled dimensions:

1. Feature representation capability, driven by advances in foundation model architectures.
2. Data-efficient learning, enabled by adaptation strategies such as parameter-efficient fine-tuning, prompt learning, few-shot learning, and domain adaptation.

This dual-perspective view highlights a fundamental research paradigm: improving segmentation performance in remote sensing requires not only stronger representation learning, but also more efficient adaptation under limited supervision. Accordingly, this section focuses on emerging architectural paradigms, while the subsequent section elaborates on corresponding adaptation strategies.

### 3.1. Transformer-Based Architectures

Transformer-based architectures [41] have become the dominant feature extraction backbone for remote sensing image segmentation. Existing Transformer variants can be divided into two representative paradigms: pure Transformer architectures and hybrid CNN–Transformer architectures. Pure Transformer models rely on self-attention to capture long-range global dependencies and have evolved from global single-scale modeling to hierarchical multi-scale modeling to fit the multi-scale and high-resolution characteristics of remote sensing imagery. In contrast, hybrid CNN–Transformer architectures integrate convolutional local inductive biases to compensate for the deficiency of pure Transformers in fine-grained local modeling and low-data generalization, and have developed diverse fusion strategies for remote sensing dense prediction tasks.

#### 3.1.1. Pure Transformer Architecture

The Vision Transformer (ViT) [3] was the first to introduce the pure Transformer architecture into computer vision, significantly reducing the strong visual inductive bias inherent in convolutional neural networks while retaining only limited spatial priors. It primarily relies on large-scale pre-training to adaptively learn visual feature representations. Compared with convolutional networks, ViT possesses a powerful global modeling capability but depends heavily on massive amounts of labeled data to achieve stable optimization. In remote sensing scenarios with limited labeled samples, ViT tends to suffer from severe overfitting, leading to a substantial decline in generalization performance. Moreover, its fixed-scale feature representation and the quadratic computational complexity of global self-attention greatly constrain the model’s applicability to high-resolution remote sensing image segmentation tasks.

To address the inherent limitations of ViT in dense prediction tasks, the Swin Transformer [7] introduces a hierarchical window-based self-attention mechanism. This model constrains attention computation within shifted windows and incorporates a patch merging operation to construct a multi-scale feature pyramid, which closely aligns with the hierarchical spatial distribution characteristics of remote sensing ground objects. This design effectively reduces computational overhead while enhancing the model’s ability to capture multi-scale spatial dependencies. Building on this foundation, Swin Transformer V2 [42] further improves training stability and high-resolution adaptability, enabling robust feature extraction from large-scale remote sensing imagery.

In summary, ViT and Swin Transformer constitute two core paradigms of remote sensing image segmentation based on pure Transformer architectures: global full self-attention modeling and hierarchical local perception modeling. Both architectures excel at capturing long-range spatial dependencies and have become mainstream backbone networks for remote sensing image segmentation tasks.

#### 3.1.2. Hybrid CNN–Transformer Architecture

Pure Transformer architectures may face challenges in preserving fine-grained details, recognizing small objects, and maintaining computational efficiency, particularly when processing high-resolution remote sensing imagery. To address these issues, hybrid CNN–Transformer architectures have emerged as a practical solution. By combining the local inductive biases and spatial locality of convolutional neural networks with the global dependency modeling capability of Transformers, these architectures aim to balance representation capacity and computational cost while potentially improving data efficiency and generalization in limited-data settings.

Based on how convolutional and Transformer modules are organized and interact, existing hybrid architectures can be broadly categorized into serial, parallel, and interleaved paradigms. Serial architectures adopt a sequential pipeline in which convolutional layers first extract local and fine-grained features, followed by Transformer modules for global context modeling. CTFuse exemplifies this pattern by placing convolutional blocks in the early encoding stages and Transformer blocks in the subsequent stages [43]. This design provides a straightforward way to combine convolutional locality with long-range dependency modeling. However, because CNN and Transformer features are primarily processed in successive stages, direct and repeated interaction between local and global representations may be limited compared with parallel or interleaved designs. Parallel architectures employ dual branches to simultaneously capture local fine-grained features and global contextual information, followed by adaptive fusion. Representative models [44,45] demonstrate strong empirical performance in remote sensing image segmentation tasks. By preserving distinct local and global representations before fusion, parallel designs can be advantageous in complex scenes and boundary-sensitive segmentation. However, the dual-branch structure generally increases architectural complexity, memory consumption, and inference cost, which may restrict its use in efficiency-critical applications. Interleaved architectures integrate convolution and self-attention operations within or across successive stages, enabling more frequent interaction between local and global features. This design can support multi-scale representation and feature coupling, but it may also increase implementation complexity and computational cost because convolutional and attention operations are repeatedly combined throughout the network. These costs can make such architectures more challenging to scale to ultra-high-resolution remote sensing imagery.

Hybrid architectures in remote sensing image segmentation exhibit distinct design trade-offs: parallel paradigms emphasize complementary feature fusion, serial designs provide a straightforward integration of local and global modeling, and interleaved approaches enable more frequent feature interaction at the cost of greater architectural and computational complexity.

From a unified perspective, Transformer-based architectures for remote sensing image segmentation can be characterized along two key design dimensions: (i) the context modeling range, from global self-attention to hierarchical or window-based attention, and (ii) the manner of locality injection, from implicit locality induced by attention constraints to explicit locality introduced by convolutions. These design choices correspond to different remote sensing challenges. Global self-attention is beneficial for long-range spatial dependency modeling, but its quadratic complexity makes direct processing of ultra-high-resolution imagery difficult and may require tiling, downsampling, or other approximation strategies. Window-based attention improves computational feasibility and multi-scale representation, although its effectiveness depends on whether local windows are sufficiently connected through shifted-window or hierarchical designs. CNN–Transformer hybrids explicitly introduce convolutional local priors, which can be beneficial for small objects, fine boundaries, and texture-rich regions, while Transformer modules complement the predominantly local receptive behavior of convolutions by modeling longer-range spatial dependencies. Therefore, Transformer-based architectures should be evaluated not only by segmentation accuracy, but also by boundary quality, scale robustness, tiling effects, cross-region transferability, and computational cost.

### 3.2. State Space Models

#### 3.2.1. Foundations and Evolution of State Space Models

As a recently emerging paradigm for sequence modeling, State Space Models (SSMs) have been increasingly introduced into remote sensing image segmentation tasks, offering an alternative perspective to Transformer-based architectures for modeling long-range dependencies. Unlike Transformers, which rely on self-attention to explicitly capture pairwise global dependencies, SSMs capture long-range dependencies through recursive state updates and theoretically exhibit linear complexity with respect to sequence length, suggesting potential scalability for high-resolution remote sensing imagery. The selective state space model represented by Mamba [8] further incorporates a content-adaptive mechanism, enhancing its expressive capacity while maintaining a favorable computational profile, which makes it potentially suitable for multi-scale and semantically complex environments. From a developmental perspective, SSMs originate from control theory. Early approaches such as S4 [46], S4D [47], and DSS [48] achieved long-sequence modeling through structured parameterization. However, their state transitions remained fixed during inference, limiting their adaptability to complex visual content. In contrast to attention-based models, SSMs and Transformers represent two fundamentally different approaches to modeling long-range dependencies: explicit pairwise interaction versus implicit state evolution, corresponding to distinct trade-offs in context modeling capacity, computational efficiency, and scalability.

To address this limitation, Mamba enhances the model’s dynamic modeling capability through an input-dependent selective mechanism, achieving a more favorable efficiency–performance trade-off. Since SSMs are inherently designed for one-dimensional sequence modeling, directly applying them to images may undermine spatial locality. To address this, Vision Mamba and its variants adopt multi-directional scanning strategies, transforming two-dimensional structures into multiple sequential paths to enhance spatial modeling.

However, such methods are essentially serialized approximations, and their performance largely depends on the scanning strategy and feature fusion approach. Currently, there is still a lack of a unified theoretical understanding. From the perspective of task adaptation, the potential advantages of Mamba in remote sensing image segmentation are mainly reflected in three aspects: (1) its content-adaptive modeling capability enables dynamic adjustment of the receptive field, allowing it to balance fine-grained structures and large-scale semantics within a unified framework; (2) its linear-complexity formulation can reduce the growth of computational cost for long sequences; and (3) its multi-directional modeling offers certain advantages for linear features with pronounced directional characteristics. These properties have contributed to the increasing adoption of SSMs in remote sensing image segmentation tasks. Nevertheless, the practical efficiency advantage of Mamba-style models over window-based attention, linear attention, and CNN–Transformer hybrids should still be interpreted cautiously, because multi-directional scanning, feature fusion, and task-specific decoding strategies may affect whether theoretical linear complexity translates into actual computational gains.

#### 3.2.2. Task-Oriented Structural Extensions for Remote Sensing Image Segmentation

Building upon these modeling characteristics, recent studies have increasingly explored how SSMs can be effectively adapted to remote sensing image segmentation tasks. Rather than forming a strictly sequential development, these efforts reflect a set of parallel explorations aimed at addressing the mismatch between sequence-based modeling and the complex spatial, structural, and multimodal characteristics of remote sensing data.

A primary line of work focuses on adapting SSMs to two-dimensional spatial modeling. Since SSMs are inherently designed for one-dimensional sequences, several studies attempt to bridge this gap through architectural modifications. For example, RSMamba [49] enhances two-dimensional modeling capability through dynamic multi-path activation, while RS3Mamba [50] introduces a VSS module as an auxiliary branch to provide global context information for the convolutional backbone. Similarly, the Samba framework [51] integrates the Mamba module into an encoder–decoder architecture, enabling end-to-end segmentation modeling. These approaches collectively demonstrate the feasibility of extending SSMs to spatially structured remote sensing tasks.

Beyond basic spatial adaptation, subsequent studies have explored improving representation quality and balancing accuracy with computational efficiency. CVMH-UNet [52] integrates Vision Mamba with multi-scale feature fusion to achieve a trade-off between accuracy and computational overhead. MCloud [53] enhances the joint modeling of long-range dependencies and local textures for cloud and cloud-shadow segmentation tasks. Other works investigate combining SSMs with linear attention mechanisms, achieving favorable efficiency–accuracy trade-offs in applications such as ocean internal wave segmentation [54]. In addition, Lite-BSSNet [55] introduces a “blueprint–bridge–repair” decoding strategy to address fragmented fine structures in high-resolution scenarios, improving structural integrity under constrained computational budgets.

In parallel, SSM-based architectures have been extended to more complex task settings and data modalities. ChangeMamba [56] introduces state space modeling into multi-temporal change detection, enhancing the characterization of spatiotemporal dependencies. Pan-Mamba [57] focuses on multimodal fusion and enables cross-channel and cross-modal information interaction in the pansharpening task. MiM-UNet [58] further strengthens multi-scale feature representation through a nested architecture, although its increased complexity poses challenges for deployment in resource-constrained scenarios. These studies suggest that SSMs can be flexibly adapted to heterogeneous remote sensing data beyond standard spatial segmentation.

Meanwhile, integrating SSMs with Transformer architectures has emerged as another important direction. MambaVision [59] combines state space modeling with the self-attention mechanism within a unified framework, demonstrating their complementarity at different modeling levels: SSMs are advantageous for long sequence modeling and may offer favorable computational profiles, while self-attention remains important for capturing fine-grained spatial dependencies. However, once self-attention is introduced, maintaining overall computational efficiency while achieving a balance between performance and complexity becomes a critical challenge in hybrid architecture design.

These studies indicate that SSM-based approaches for remote sensing segmentation are evolving through diverse and largely parallel adaptations rather than a simple linear progression. Despite encouraging results, several issues remain. The multi-directional scanning strategy still lacks unified theoretical analysis, and its relationship with the spatial structure of remote sensing imagery remains unclear. Direct comparisons among SSMs, window-based attention, linear attention, and CNN–Transformer hybrids are also limited, and whether theoretical linear complexity translates into practical efficiency gains depends on task characteristics and implementation details. In addition, existing evaluation settings remain constrained in terms of sensor diversity, geographic coverage, and resolution variability, leaving cross-domain generalization insufficiently validated. Taken together, these limitations suggest that while SSMs provide a promising alternative to attention-based architectures, their role in remote sensing segmentation is still evolving, particularly with respect to theoretical grounding, spatial inductive bias, and integration with heterogeneous data modalities.

### 3.3. Prompt-Driven General Segmentation Models

Prompt-driven segmentation has emerged as a new paradigm for general-purpose image segmentation, exemplified by the Segment Anything Model (SAM) proposed by Meta AI in 2023. SAM aims to achieve universal image segmentation through large-scale pre-training. Its architecture comprises three main components: an image encoder based on a pre-trained Vision Transformer (ViT) backbone for extracting high-dimensional features; a prompt encoder that processes both sparse spatial prompts (e.g., points and bounding boxes) and dense prompts; and a lightweight Transformer decoder that integrates image features and prompts via bidirectional attention to generate segmentation masks. This paradigm shifts segmentation from a fixed feed-forward prediction task toward a prompt-conditioned and interactive process, effectively introducing a new interface between human guidance and model inference, and extending representation-centric modeling toward interaction-driven settings.

This promptable paradigm enables human-in-the-loop interactive segmentation and provides a flexible starting point for remote sensing image segmentation. SAM is trained on the SA-1B dataset, which contains over 11 million images and 1.1 billion masks, and adopts a human–machine collaborative data engine with a closed-loop design to iteratively improve model performance. In remote sensing image segmentation, the original SAM shows potential cross-domain transferability, particularly in capturing structural cues such as building boundaries, road networks, and vegetation textures when appropriate prompts are provided. However, this capability should be interpreted cautiously. Direct zero-shot use of the original SAM remains constrained by its category-agnostic mask formulation and reliance on manual prompts, which limit its scalability in large-scale automated remote sensing segmentation. In addition, domain discrepancies between natural image pre-training and remote sensing imagery, including overhead viewing geometry, high feature density, and non-RGB spectral characteristics, may affect its performance on small objects, weak boundaries, and heterogeneous sensor modalities.

These challenges further emphasize the importance of adaptation strategies, particularly prompt engineering and parameter-efficient fine-tuning, which will be reviewed in Section 4.

### 3.4. Self-Supervised and Multimodal Pre-Training Models

While the previous subsections focus on how representations are structured by architectural design, this subsection shifts the focus to how they are learned from data. Pre-training therefore plays a central role in shaping the representation space of foundation models, beyond the choice of architectural backbones.

Recent research in remote sensing foundation models has evolved along three major paradigms: self-supervised visual representation learning, contrastive learning and vision–language pre-training [11,60], and masked image modeling. These paradigms go beyond pure visual feature extraction and instead provide different ways of constructing representation spaces, including invariant feature learning, cross-modal semantic embedding, and reconstruction-based representation learning. In practice, the main differences lie in how they balance semantic abstraction, data efficiency, and flexibility.

#### 3.4.1. Self-Supervised Visual Representation Learning (DINO Family)

Self-supervised visual representation learning, exemplified by the DINO family, is a paradigm based on self-distillation and invariance learning. By aligning representations across global and local views without manual annotations, these methods learn structured and object-centric representations from large-scale unlabeled data.

DINO (Self-Distillation with NO labels) [9] demonstrates strong representation learning capability through global–local view alignment. However, its fixed-scale cropping strategy is suboptimal for the inherent multi-scale variability of remote sensing imagery. DINO-MC [61] addresses this limitation by introducing multi-sized local cropping, enabling more effective capture of hierarchical semantic structures across spatial resolutions.

DINOv2 [62] further advances this paradigm through large-scale data curation and knowledge distillation, significantly improving representation quality and cross-domain generalization. Building upon this foundation, GeoSA-BaSA integrates parameter-efficient fine-tuning with geospatial-aware batch self-adaptation and a Mask2Former segmentation head, achieving strong performance across diverse remote sensing datasets [63]. Additional studies show that LoRA-based [64] lightweight fine-tuning enables DINOv2 to maintain competitive performance under limited supervision, particularly in geological image analysis tasks.

More recently, the DINOv3 technical report [65] further explores large-scale self-supervised visual representation learning with billion-parameter Vision Transformer backbones and introduces Gram Anchoring regularization to mitigate dense-feature degradation during long training schedules. Although DINOv3 provides useful evidence on the scaling trend of self-supervised vision foundation models, its effectiveness specifically for remote sensing semantic segmentation still requires further task-specific validation under heterogeneous sensors, resolutions, and annotation settings.

To provide a clearer perspective on the evolution of the DINO family, Table 3 summarizes the key differences across DINOv1, DINOv2, and DINOv3 in terms of training objectives, model scaling, architectural design, and representation characteristics.

The effectiveness of the DINO family can be attributed to its emergent object-centric representations, where feature components naturally align with object boundaries and part-level structures without explicit supervision. This property supports unsupervised segmentation and robust cross-domain transfer. Beyond standard 2D image understanding, DINO-based representations have been extended to more complex scenarios. For instance, integrating normalized digital surface model elevation data helps reduce semantic ambiguity in complex terrain and improves segmentation accuracy [66,67]. Multi-layer feature aggregation further enhances robustness across heterogeneous platforms such as satellite and UAV imagery [68]. Moreover, projecting DINO features into 3D space enables high-precision point cloud segmentation, extending its applicability to multimodal fusion tasks [69].

Beyond purely visual representation learning, recent extensions have incorporated cross-modal grounding to enhance semantic flexibility. Grounding DINO extends the original framework to open-vocabulary detection via text-guided grounding [70]. However, its dual-branch design introduces significant computational overhead, motivating the development of lightweight cross-modal modules and optimized neck architectures to improve efficiency [71]. Building upon this, integrating Grounding DINO with segmentation pipelines enables effective zero-shot interpretation. For example, multi-stage preprocessing strategies, including tiling and statistical refinement, significantly improve segmentation accuracy [72]. Further extensions such as GDPGO-SAM combine prompt optimization with open-set perception, enabling fine-grained zero-shot segmentation of typical land-cover objects [73].

Despite their strong generalization ability, DINO-based methods fundamentally rely on visual invariance learning. From a comparative perspective, DINO-based methods excel in learning semantically meaningful and transferable representations with relatively high data efficiency, but remain limited in cross-modal expressiveness and semantic controllability.

#### 3.4.2. Vision–Language Pre-Training (CLIP-Based Models)

Contrastive vision–language pre-training is a paradigm centered on cross-modal semantic alignment. Models such as CLIP map images and text into a shared embedding space, enabling open-vocabulary recognition based on textual descriptions. This improves semantic flexibility, allowing remote sensing models to generalize beyond fixed category sets.

Direct transfer of CLIP to remote sensing is hindered by substantial domain discrepancies, including differences in viewing geometry, spectral characteristics, and semantic granularity. These discrepancies lead to misalignment between visual and textual representations, limiting downstream performance.

To mitigate these issues, various adaptation strategies have been proposed. RemoteCLIP [74] constructs domain-specific datasets using Box-to-Caption and Mask-to-Box transformations, improving cross-modal alignment while introducing unavoidable annotation noise. Other approaches include knowledge graph-enhanced semantic modeling (KG-OVRSeg [75]), implicit prior decoupling for few-shot segmentation [76], and multi-granularity context fusion for instance-level tasks [77].

Despite these advances, most methods rely on post hoc adaptation rather than addressing the root cause of domain misalignment during pre-training. As a result, performance remains sensitive to cross-modal supervision quality and alignment consistency, highlighting a fundamental limitation of current vision–language models in remote sensing. Compared with invariant feature learning, vision–language models offer stronger semantic flexibility but rely more heavily on high-quality cross-modal supervision, making their data efficiency and stability more sensitive to domain shifts.

#### 3.4.3. Masked Image Modeling (MAE-Based Methods)

Masked image modeling focuses on reconstruction-based self-supervision, where models learn latent representations by reconstructing masked inputs. The Masked Autoencoder (MAE) [10] has demonstrated strong performance in natural image domains, but its direct application to remote sensing is limited by mismatches between pre-training objectives and data characteristics.

Specifically, random masking strategies fail to align with multi-scale object distributions, while reconstruction objectives tend to emphasize low-level pixel fidelity rather than high-level semantic abstraction. This leads to suboptimal transferability to downstream segmentation tasks.

To address these limitations, existing extensions can be interpreted along several complementary directions. FG-MAE [78] enhances spectral–spatial reconstruction to improve robustness for SAR imagery. SatMAE [79] adapts masked autoencoding to temporal and multispectral satellite imagery by introducing temporal embeddings, spectral positional encodings, and independent masking strategies across temporal or spectral dimensions. A^2^-MAE [80] introduces anchor-aware masking and geographic encoding to better model spatial dependencies. Scale-MAE [81] captures cross-scale correlations by reconstructing both low-frequency and high-frequency components, enabling robust multi-scale representation.

Despite these improvements, most approaches still emphasize individual aspects such as spectral–spatial reconstruction, temporal or multispectral modeling, geographic encoding, or scale awareness, and a unified framework that jointly models semantic abstraction, scale variation, and spatio-temporal consistency remains insufficiently developed. Although masked modeling provides a flexible and scalable pre-training strategy for remote sensing imagery, it tends to favor reconstruction fidelity over semantic abstraction. Therefore, its effectiveness for downstream segmentation still depends on additional adaptation mechanisms, especially when fine-grained boundary prediction, multi-scale objects, and heterogeneous sensor characteristics are involved.

#### 3.4.4. Unified and Hybrid Pre-Training Paradigms

Recent research increasingly explores hybrid paradigms that integrate invariance learning (DINO), cross-modal alignment (CLIP), and reconstruction-based modeling (MAE). These approaches aim to jointly leverage complementary inductive biases to improve representation generality.

For example, combining DINO with CLIP enhances the integration of structural visual priors and open-vocabulary semantics, improving local–global consistency. Similarly, integrating masked modeling with state space architectures provides efficient solutions for long-sequence and multi-temporal data processing.

Beyond single-modality learning, multi-source heterogeneous fusion has become a key direction. Hybrid modality MAE frameworks jointly model SAR and optical data through dual-task reconstruction, improving cross-modal feature integration [82]. SenPa-MAE [83] further incorporates physical sensor parameters such as spectral response and spatial resolution to enable sensor-agnostic learning.

Large-scale foundation models such as SkySense and SkySense++ [84,85] extend this paradigm by integrating multimodal and spatio-temporal learning across diverse Earth observation tasks. However, even unified pre-training frameworks cannot fully eliminate the intrinsic conflicts between invariance, alignment, and reconstruction objectives. As a result, the burden of resolving these conflicts is often shifted to downstream adaptation and fine-tuning stages. In practice, these models suffer from high computational cost and strong dependence on accurate spatio-temporal registration. The impact of misalignment errors on fusion performance remains insufficiently explored. More broadly, data and computational fragmentation continues to limit large-scale deployment and cross-task generalization [86].

These paradigms embody three distinct learning principles: invariance learning, cross-modal alignment, and reconstruction-based modeling. While each paradigm excels in specific aspects, a fundamental tension persists between semantic abstraction and reconstruction fidelity, as well as between closed-set representation learning and open-vocabulary generalization.

Current pre-training paradigms remain inherently fragmented. Despite their complementary strengths, none can simultaneously address the challenges of multi-scale representation, cross-modal alignment, and spatio-temporal consistency. Such limitations are not only architectural but persist even after pre-training, creating a gap between general-purpose representations and domain-specific requirements in remote sensing. Consequently, adaptation and fine-tuning strategies become essential for reconciling these discrepancies and enabling robust generalization across heterogeneous scenarios.

**Figure 4:**
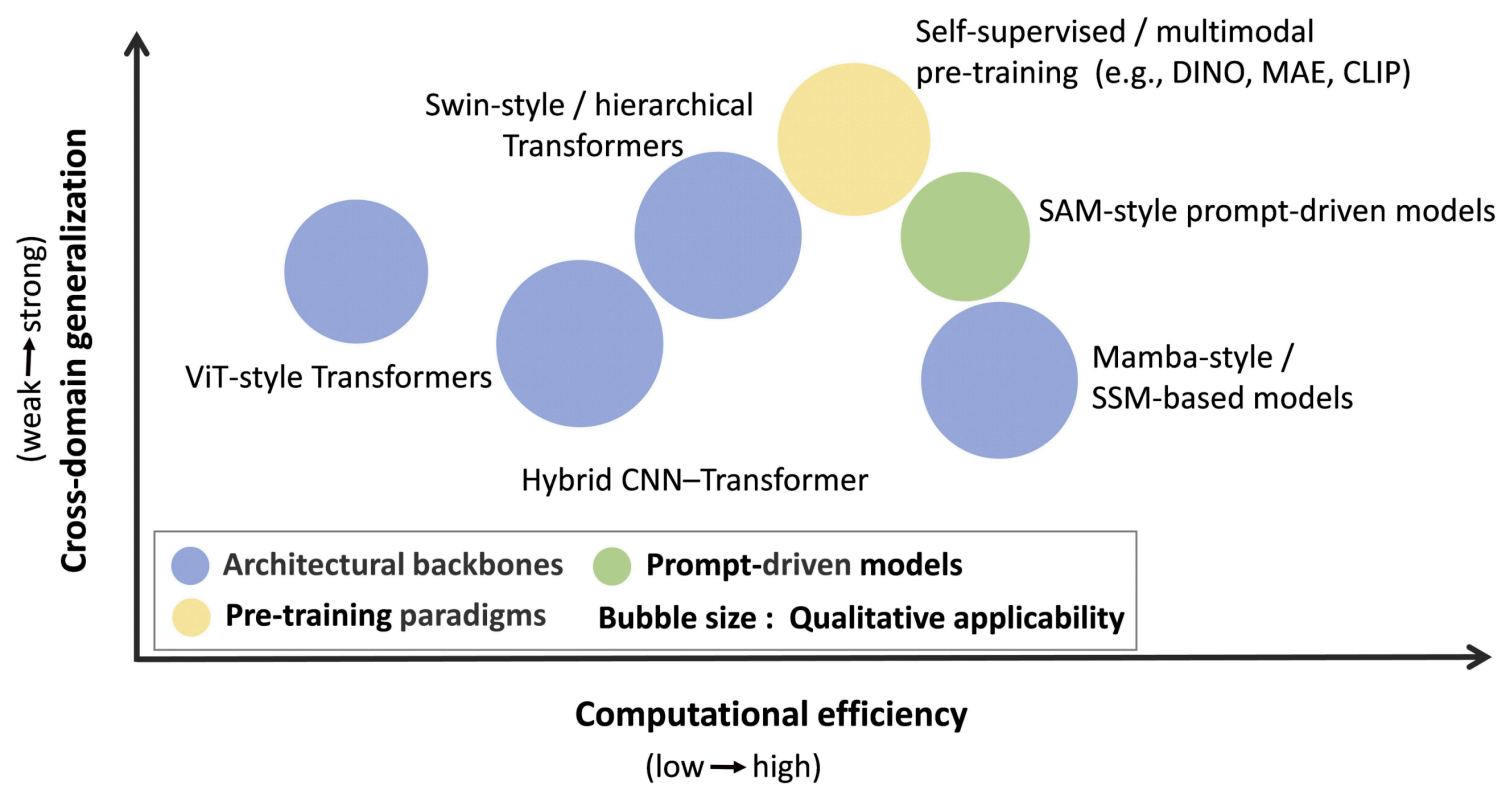
Conceptual positioning of major foundation-model paradigms for remote sensing image segmentation. The axes and bubble sizes are heuristic visual cues for the qualitative trade-offs discussed in Section 3, rather than a quantitative benchmark ranking of specific models.

Based on the above discussion, Figure 4 provides a conceptual positioning of the major foundation-model paradigms reviewed in this section. Rather than ranking specific models under a unified benchmark, the figure uses heuristic visual cues to summarize broad qualitative tendencies in computational scalability, transferability, and segmentation applicability. This conceptual view helps explain why no single paradigm can fully satisfy the requirements of remote sensing image segmentation across heterogeneous sensors, resolutions, and task settings, thereby motivating the adaptation and fine-tuning strategies discussed in Section 4.

## 4. Adaptation and Fine-Tuning Strategies

### 4.1. Parameter-Efficient Fine-Tuning (PEFT)

As the scale of foundation models continues to expand, the computational and storage costs of full fine-tuning have become increasingly prohibitive. Parameter-efficient fine-tuning methods achieve efficient domain adaptation by freezing most of the pre-trained parameters and updating only a small subset. These methods have demonstrated significant potential in remote sensing image segmentation. This section discusses the topic from two perspectives: the low-rank decomposition strategy and the combination of adapters with prompt learning.

#### 4.1.1. Adaptation Strategy Based on Low-Rank Decomposition

**Figure 5:**
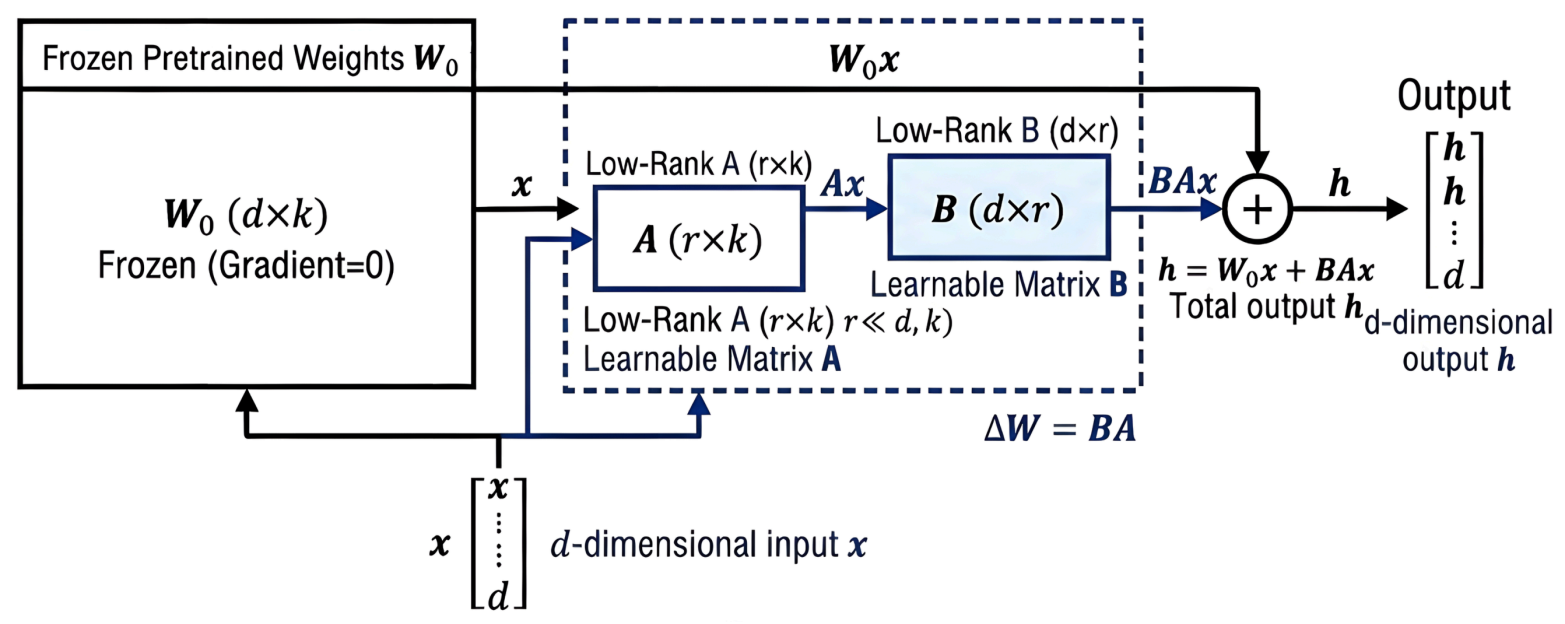
Illustration of the LoRA mechanism for parameter-efficient fine-tuning. LoRA keeps the pre-trained weight matrix frozen and approximates parameter updates through two learnable low-rank matrices, thereby reducing the number of trainable parameters and computational overhead while preserving the original backbone structure. Black arrows denote the frozen pre-trained path, whereas blue arrows denote the learnable low-rank update path.

The low-rank decomposition strategy is built upon the observation that weight updates during fine-tuning often exhibit a low intrinsic rank. By introducing learnable low-rank matrices to approximate parameter updates while keeping the pre-trained weights frozen, this approach significantly reduces the number of trainable parameters and computational overhead while often maintaining competitive performance [64]. Representative methods such as Low-Rank Adaptation (LoRA) have emerged as a prominent approach for remote sensing image segmentation. As illustrated in Figure 5, LoRA decomposes parameter updates into two learnable low-rank matrices while keeping the original pre-trained weights frozen.

Despite its effectiveness, standard LoRA inherits the structural biases of Transformer architectures, where linear projection layers lack strong translation invariance and explicit local inductive bias. This limitation is particularly pronounced in remote sensing, where fine-grained textures and spatial locality are critical. Consequently, naive low-rank updates alone are often insufficient to bridge the domain gap between natural image pre-training and remote sensing data. To mitigate this issue, a targeted extension is to introduce local inductive biases into low-rank adaptation, aiming to improve the spatial modeling capability of LoRA-style methods for dense prediction tasks.

Conv-LoRA [87] introduces lightweight convolutional modules between the encoder and decoder, effectively embedding image-specific inductive biases into the Vision Transformer backbone. To further accommodate multi-scale characteristics, it incorporates a Mixture-of-Experts design with multiple parallel convolutional experts, each specializing in a different feature scale. A gating network dynamically selects experts during inference, improving performance in multi-domain segmentation tasks. This added flexibility comes at the cost of increased optimization complexity: the gating mechanism may introduce sensitivity to batch size and learning rate scheduling, and the selection of expert numbers and scale coverage still depends on manual tuning to some extent. Its robustness under large cross-domain scale variations remains insufficiently evaluated in the current literature.

In parallel, lightweight deployment has emerged as another important direction. By freezing most parameters and restricting adaptation to LoRA modules, efficient domain adaptation can be achieved even on resource-constrained devices. For instance, Wasil et al. [88] demonstrate effective forest ground segmentation in drone imagery, while PriorSAM-DBNet [89] employs a dual-branch architecture combining a Swin Transformer with a frozen SAM backbone to enhance structural prior modeling. Notably, only 0.91 M parameters are fine-tuned, yet strong performance is achieved in complex urban scenes. These lightweight designs inevitably reduce model capacity, especially when scene complexity exceeds the representational limit of the adapted parameters, leading to potential performance saturation that remains underexplored and poorly quantified in existing studies.

Related studies also suggest the potential of LoRA for multimodal remote sensing tasks. For example, a two-stage LoRA fine-tuning strategy [90], built upon Qwen3-VL, inserts LoRA into both the visual and text encoders to support cross-modal representation learning and few-shot adaptation. This design provides useful evidence for how LoRA can be used to adjust vision–language foundation models under limited remote sensing annotations. However, since existing evaluations are mainly conducted on object detection rather than pixel-level segmentation, its relevance to remote sensing semantic segmentation remains indirect and requires further validation. In addition, the strategy assumes that visual-side LoRA features learned from base categories can generalize to novel categories, which may be challenged by large domain shifts between natural and remote sensing imagery.

Beyond static image-based tasks, LoRA-based adaptation has also been explored in remote sensing video segmentation. ROS-SAM [91] applies LoRA-based fine-tuning to continuous-frame remote sensing segmentation and tracking scenarios. By combining LoRA-based fine-tuning with an optimized mask decoder, it enhances both global context modeling and boundary refinement. The redesigned data pipeline enables scale-aware training and high-quality single-object prediction during inference. However, its performance relies on stable frame rates and temporal continuity, rendering it sensitive to occlusion and irregular sampling. Such robustness remains insufficiently validated, as existing evaluations mainly concentrate on moving object scenarios while leaving its applicability to broader static or multi-class segmentation settings unclear.

These extensions suggest that low-rank adaptation is gradually moving beyond simple parameter-efficient tuning and is increasingly used to introduce task-specific structures into foundation models for remote sensing segmentation and related visual understanding tasks.

#### 4.1.2. Based on the Structured Adapter Fine-Tuning Strategy

**Figure 6:**
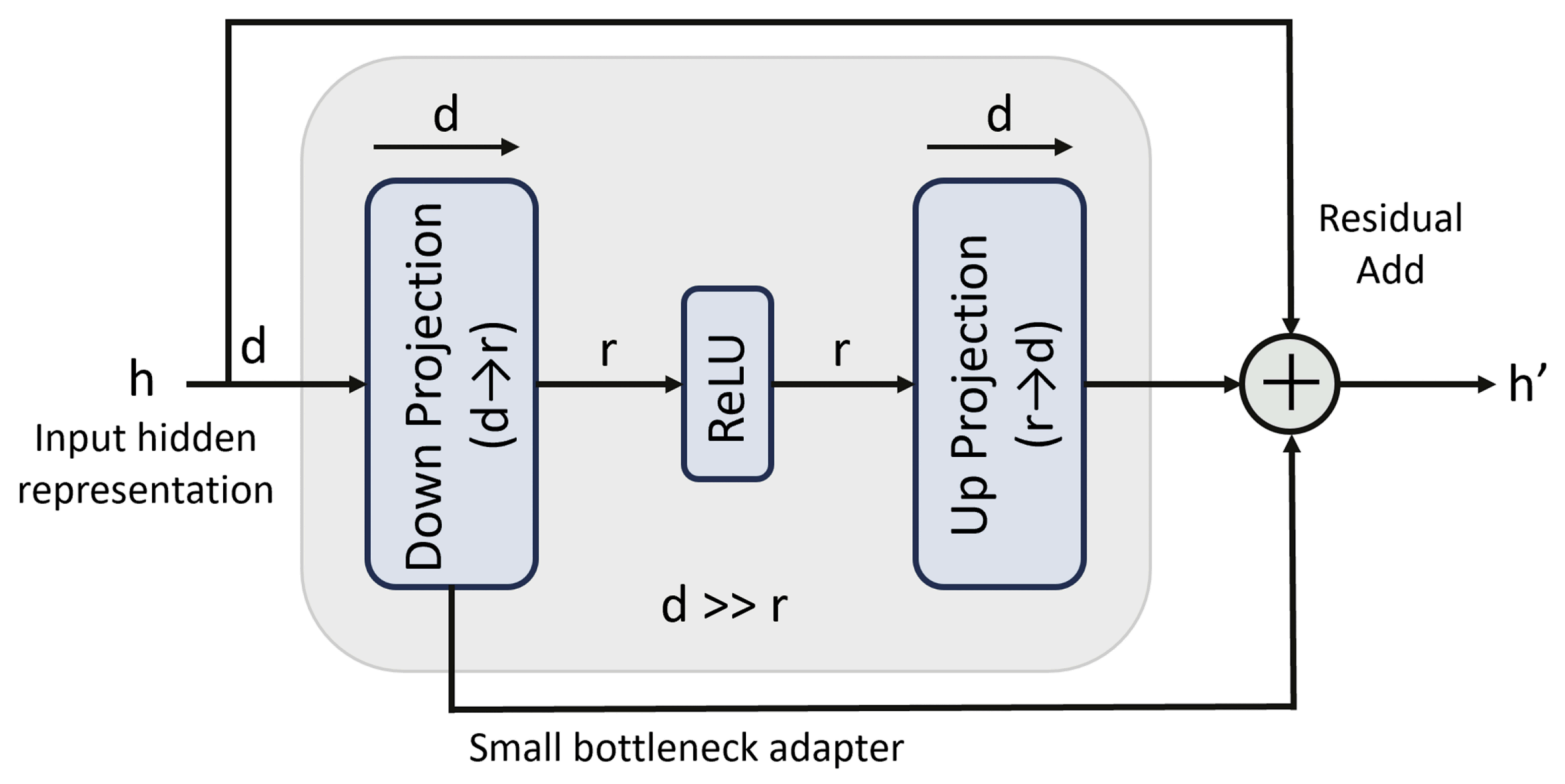
Illustration of the adapter mechanism for structured fine-tuning. The adapter module introduces a lightweight bottleneck structure consisting of down-projection and up-projection layers with residual connections, enabling task-specific feature adaptation while keeping the backbone network frozen.

Unlike LoRA, which implicitly adjusts weight matrices through low-rank decomposition, adapter-based methods explicitly introduce task-specific feature transformations by inserting bottleneck MLP modules into Transformer blocks while keeping the backbone network frozen [92]. As illustrated in Figure 6, the adapter module performs feature adaptation through a lightweight down-projection and up-projection bottleneck structure. This explicit structural intervention provides improved controllability and interpretability, which is particularly beneficial for handling the multimodal heterogeneity of remote sensing data, such as SAR imagery and scenarios involving complex multi-scale feature interactions. However, unlike LoRA-style reparameterization, these additional modules cannot be merged into backbone weights during inference, resulting in non-negligible computational overhead. Key hyperparameters, including bottleneck dimension and insertion position, remain highly sensitive to performance and are typically determined through empirical tuning rather than principled guidance derived from the intrinsic characteristics of remote sensing data.

Building on this basic mechanism, existing studies extend adapter-based adaptation along several complementary directions.

SAM-Adapter [93] follows a straightforward strategy by directly modifying internal feature representations and integrates lightweight adapters and domain prompts into the ViT encoder of SAM, enabling efficient transfer to downstream tasks while preserving the generalization capability of the pre-trained model. Its effectiveness across tasks such as camouflaged object detection, shadow detection, and remote sensing building extraction demonstrates that explicit feature transformation can help bridge the distribution gap between general-purpose foundation models and domain-specific data. The reliance on manually designed domain prompts implies that task-relevant prior knowledge must be explicitly encoded, which may limit scalability when adapting to diverse sensor types and multi-class semantic segmentation scenarios.

Rather than altering internal representations, some approaches exploit SAM as an external knowledge source. The SAM-assisted framework [94], for example, incorporates object and boundary information generated by SAM into downstream segmentation models as auxiliary supervision signals. By designing object consistency and boundary preservation loss functions, it enables the effective utilization of SAM’s zero-shot object recognition capability without modifying the backbone architecture, thereby improving compatibility with different segmentation networks. Its effectiveness is strongly tied to the quality of pseudo-labels, making it sensitive to noise in complex scenes. In complex remote sensing scenarios with blurred boundaries or densely distributed objects, inaccurate predictions may introduce noisy supervision signals. In addition, the inclusion of auxiliary loss terms introduces additional hyperparameters for loss balancing, increasing optimization complexity and potentially affecting convergence stability.

Further improvements can be achieved by refining the optimization process through task-specific supervision design. SAM2MS [95] employs cascaded subtraction blocks to process adjacent feature maps and introduces a parameter-free supervision network based on a pre-trained ResNet50 to impose multi-level constraints, including background suppression, contour refinement, and region-of-interest consistency. This design enhances optimization interpretability and improves boundary delineation. It also achieves strong performance on road-extraction benchmarks, including the DeepGlobe road-extraction dataset [17], SpaceNet road-network data [16], and Massachusetts Roads [15], while maintaining cross-dataset generalization without additional training. The introduction of auxiliary supervision networks increases system complexity and may affect convergence stability. Moreover, the stability of multi-level constraints under limited annotation conditions remains insufficiently validated, and current evaluations are largely restricted to road extraction tasks, leaving their applicability to more general segmentation scenarios unclear.

Adapter-based designs can also be tailored to address modality-specific characteristics. CWSAM [96] targets SAR imagery by introducing class-aware adapters and redesigning the mask decoder to support multi-class semantic segmentation. It further incorporates a task-specific multilayer perceptron (MLP) module to inject low-frequency information, aiming to mitigate the effects of speckle noise and complex scattering patterns. This design is well-suited for SAR imagery while implicitly emphasizing low-frequency representations, whose role in semantic discrimination remains insufficiently validated in scenarios dominated by high-frequency structural patterns, such as dense urban environments. In addition, the modified decoder departs from SAM’s original prompt-driven paradigm, reducing compatibility with existing interaction mechanisms.

Effective adaptation also requires explicit modeling of multi-scale representations within the adapter structure. MSA-SAM [97] adopts a dual-adapter design by embedding a Pyramid Multi-scale Adapter in global attention layers and a Window Multi-scale Adapter in local attention layers. By combining multi-ratio pooling with cross-attention for global context modeling and employing variable window sizes for local feature extraction, the model enables effective multi-scale feature fusion. Experimental results on benchmarks such as Vaihingen [14], Potsdam, and UAVid [19] demonstrate competitive performance with relatively few trainable parameters. The coexistence of multiple adapter modules increases architectural complexity and introduces additional hyperparameter sensitivity. In addition, hyperparameter configurations, including pooling ratios and window sizes, are highly dataset-dependent, and their transferability across datasets remains insufficiently explored. Current evaluations are also primarily limited to optical imagery, leaving adaptability to heterogeneous modalities such as SAR and multispectral data an open question.

Compared with LoRA, adapter-based designs provide a more explicit way to reshape intermediate representations, which is particularly useful for handling modality-specific characteristics. This advantage, however, comes with increased architectural complexity and a stronger dependence on empirical configuration. To further clarify their differences, Table 4 summarizes LoRA- and adapter-based PEFT strategies in terms of adapted components, parameter and inference cost, benchmark evidence, and main limitations. Since existing studies use different backbones, datasets, and evaluation protocols, the table emphasizes representative trends rather than direct leaderboard-style ranking.

### 4.2. Prompt Engineering and Context Learning

#### 4.2.1. Prompt Engineering Strategies for SAM

The effectiveness of the SAM in general vision tasks largely stems from its flexible interactive prompt mechanism, including points, bounding boxes, and coarse masks. In large-scale remote sensing scenarios, such manual interaction introduces significant efficiency bottlenecks, making it unsuitable for automated deployment. This limitation has motivated increasing efforts toward automated prompt generation, aiming to enable SAM to operate without human intervention. Prompt automation in remote sensing remains non-trivial due to dense object distribution, scale variation, and weak boundary separability.

To reduce reliance on manual interaction, prompts can be derived directly from visual representations. RSPrompter [98] introduces a Prompt Learning Mechanism, where a lightweight feature enhancer and a prompt generator extract multi-scale features from intermediate layers of the SAM encoder to produce category-specific prompt embeddings. This design demonstrates the feasibility of automatic prompting, but its reliance on a Region Proposal Network limits robustness in object-dense and boundary-ambiguous scenarios. In addition, the introduction of auxiliary modules increases training complexity and computational overhead. RSAM-Seg [99] explores parameter-efficient adaptation by incorporating Adapter-Scale and Adapter-Feature modules. The former enhances feature representation within ViT blocks through a bottleneck transformation, while the latter integrates FFT-based high-frequency edge information with image embeddings to guide prompt generation. This design outperforms conventional segmentation models such as U-Net [100], DeepLabV3+ [101], and SegFormer [102], with its effectiveness heavily dependent on the reliability of high-frequency feature extraction. In low-contrast or texture-homogeneous regions, frequency-domain representations may provide limited structural cues, which in turn affect the quality of the generated prompts. Moreover, the scalability of such adapter-based designs has not yet been extensively validated on ultra-large-scale remote sensing imagery.

In some cases, prompt construction is further integrated into the segmentation process itself. RSPS-SAM [103] removes manual prompts by introducing a Batch Attention Pyramid to capture multi-scale and long-range contextual information across cropped patches, mitigating the loss of global context in large-scale scenes. Its mask-based decoder jointly performs target perception and category prediction, effectively embedding prompt generation into the segmentation pipeline. This unified design eliminates standalone prompt generation overhead, with performance constrained by the quality of early-stage feature representations. In scenarios involving dense objects or highly similar backgrounds, ambiguities in early-stage feature extraction may propagate through the model, leading to unreliable segmentation outputs. Furthermore, its evaluation is currently limited to a single dataset, leaving its generalization capability across diverse remote sensing conditions unclear.

Multimodal strategies further extend this idea by incorporating textual information. RS2-SAM2 [104] adopts an end-to-end joint encoding framework that simultaneously processes visual and textual inputs to produce aligned multimodal embeddings and prompt tokens. A bidirectional hierarchical fusion module further strengthens cross-modal interaction, reducing the dependency on intermediate predictions. This design supports referring remote sensing image segmentation by adapting SAM2 to text-guided target localization and mask generation. Nevertheless, such approaches remain sensitive to the quality and consistency of textual descriptions, suggesting that sufficiently strong or explicit alignment constraints between visual and textual embeddings may still be lacking. In addition, their applicability has mainly been demonstrated in referring image segmentation, and their effectiveness in general semantic segmentation settings remains uncertain.

These developments suggest a gradual shift from the original interactive use of SAM toward more automated and integrated designs, where prompt generation is learned from visual representations, embedded into the segmentation pipeline, or combined with text-guided semantic grounding. To avoid treating these methods as isolated cases, Table 5 summarizes the major SAM usage and adaptation modes discussed across this review, including the original prompt-driven SAM baseline, automated prompt generation, parameter-efficient SAM adaptation, and text-guided SAM pipelines. This comparison highlights their different prompt requirements, adapted components, remote-sensing targets, and practical limitations.

#### 4.2.2. Context Learning and Instruction Fine-Tuning for MLLMs

As prompt engineering gradually extends from visual models to multimodal settings, MLLMs provide a more flexible interface for remote sensing image understanding. Instead of relying on structured prompts such as points or bounding boxes, these models leverage natural language instructions to guide visual perception, enabling a unified framework that integrates reasoning, description, and segmentation. Adapting such models to remote sensing, however, requires more than directly transferring general-purpose architectures.

One line of work focuses on enabling pixel-level grounding within MLLMs, thereby allowing language instructions to be translated into dense spatial predictions. GeoPix [105] exemplifies this direction by introducing a Mask Predictor that maps visual encoder features into segmentation masks conditioned on segmentation token embeddings derived from the language model. To address the scale variability of remote sensing objects, it further incorporates a Class-Aware Learnable Memory module to store category-specific contextual representations. A two-stage training strategy is adopted to decouple text generation from mask prediction, alleviating optimization conflicts in multi-task learning. This design demonstrates the feasibility of language-guided segmentation, but its reliance on dataset-level category statistics constrains flexibility in open-set scenarios. In addition, the staged optimization procedure increases training complexity, and the scale of the GeoPixInstruct dataset may still limit generalization across diverse environments.

While GeoPix illustrates how language instructions can be connected to pixel-level mask prediction, the evaluation of remote sensing MLLMs also requires benchmarks that cover broader perception and reasoning capabilities. In parallel with model design, benchmarking has become an important component in evaluating remote sensing MLLMs. XLRS-Bench [106] provides a benchmark covering a wide range of perception and reasoning tasks, enabling more structured performance comparison. By organizing tasks into multiple categories of multimodal understanding and reasoning, it supports more fine-grained analysis of model capabilities. Its current focus is still biased toward general vision–language abilities, with relatively limited coverage of domain-specific challenges such as temporal change analysis and multi-source data fusion. In addition, potential geographical bias in the data distribution may affect the reliability of cross-region evaluation.

These works point toward a language-driven paradigm, where high-level instructions replace explicit prompts to guide visual perception in remote sensing. Overall, MLLM-based instruction tuning and SAM-centered prompt engineering represent two complementary interface-level adaptation strategies: the former relies mainly on natural-language instructions, whereas the latter uses structured visual prompts. While MLLM-based instruction tuning can provide greater semantic flexibility and reasoning capability, it remains constrained by instruction-data quality, cross-modal alignment, and limited pixel-level evaluation.

### 4.3. Zero-Shot and Few-Shot Learning

#### 4.3.1. Meta-Learning and Prototype Network Strategies for Few-Shot Semantic Segmentation

Few-Shot Semantic Segmentation (FSS) enables rapid adaptation to new categories by learning from a limited number of annotated samples, providing an effective solution for remote sensing image segmentation under scarce supervision. However, remote sensing imagery differs substantially from natural images, exhibiting densely distributed objects, significant intra-class variation, and pronounced scale diversity. These characteristics impose stricter requirements on contextual modeling and inter-class discrimination for FSS models [107]. As a result, recent work has approached this problem from multiple perspectives, leading to a diverse set of methodological developments.

A central line of work focuses on improving prototype learning, which underpins most FSS frameworks. Peng et al. [108] proposed a Multi-granularity Aggregation Network that captures semantic correspondences between support–query pairs across multiple spatial scales, enhancing adaptability to multi-scale land cover objects. Building on this idea, Liu et al. [109] introduced a Selective Prototype Aggregation method that adaptively combines support prototypes with self-support representations derived from the query set, alleviating biases caused by limited support diversity and base-class interference. Despite improved prototype quality, these methods remain fundamentally grounded in pixel-level aggregation. In remote sensing scenarios characterized by ambiguous boundaries and visually similar backgrounds, such representations may still lack sufficient discriminative power. Moreover, multi-scale aggregation strategies introduce considerable computational overhead as the number of scales increases, posing challenges for deployment in resource-constrained applications.

Beyond prototype construction, a related direction focuses on modeling feature associations between support and query samples more effectively. Wang et al. [110] adopted a dual-branch feature enhancement architecture, where hierarchical feature processing and adaptive interaction enable more accurate prototype estimation and pixel-level matching. He et al. [111] further extended this idea by combining convolutional operations with deformable Transformers to build a hierarchical relation learning framework that captures semantic dependencies from local to global levels, demonstrating the effectiveness of hierarchical relation modeling on large-scale remote sensing datasets. These studies suggest that relation-aware feature interaction can complement prototype construction by improving support–query matching. However, their effectiveness still depends on the robustness of feature correspondence. In remote sensing images with ambiguous boundaries, visually similar backgrounds, or large intra-class variations, unstable correspondences may still lead to unreliable segmentation results.

Generalized few-shot semantic segmentation (GFSS) extends the standard setting by requiring models to maintain performance on base classes while recognizing novel categories. Gao et al. [112] proposed the FoMA framework, which integrates zero-shot predictions from foundation models with few-shot learning through support label enrichment, general knowledge distillation, and a voting fusion of experts strategy. This design highlights the potential of leveraging foundation model priors in GFSS. Li et al. [113] developed the SegLand framework for land cover mapping, combining multiple base learners with an improved projection onto an orthogonal prototype network to enhance base-class recognition while preserving spatial continuity and semantic consistency. The effectiveness of these methods depends on underlying assumptions that may not hold in complex remote sensing scenes. In FoMA, the reliability of expert voting is closely tied to the quality of zero-shot predictions, which can degrade under significant domain shifts. In SegLand, the assumption of well-separated category prototypes is often violated in remote sensing scenes where semantically similar categories such as grassland and farmland often exhibit overlapping feature distributions.

In parallel with methodological advances, benchmark evaluation has also received increasing attention. The OpenEarthMap GFSS benchmark [35,114] provides a unified platform for assessing model performance in remote sensing scenarios. Its coverage remains limited in terms of geographic diversity and sensor types, raising concerns about the generalizability of evaluation results. Beyond benchmarking, recent work has explored more unified modeling paradigms. Immanuel et al. [115] proposed a learnable prompt-based method built upon SegGPT, where independent prompts are optimized for each category and combined with patch-based prediction and stitching strategies to handle scale variation and boundary discontinuities. Bi et al. [116] introduced AgMTR, which constructs semantic relationships through adaptive agent mining at a local level, offering an alternative representation for capturing spatial dependencies.

Vision–language priors and multimodal information have also been explored to enhance few-shot segmentation performance. Jiang et al. [76] proposed ICPD-Net, which identifies foreground–background semantic confusion in CLIP pre-training and addresses it through a prior disentanglement mechanism, achieving consistent performance gains across multiple datasets. Li et al. [117] developed the Mask-Guided Correlation Learning method, which improves support–query matching through over-segmentation mask guidance and explicit modeling of foreground–background relationships, showing particular advantages for small and elongated targets. Despite these advances, a fundamental challenge remains: the pre-training distribution of vision–language models differs substantially from remote sensing data in imaging perspective, spectral characteristics, and semantic granularity. Characterizing this domain gap and designing principled transfer strategies remain largely underexplored.

These developments reflect a shift from prototype-centric matching toward more flexible formulations that incorporate relational modeling, generalized few-shot settings, and external priors, aiming to improve adaptability to complex remote sensing scenes.

#### 4.3.2. Zero-Shot Learning and Open-Vocabulary Semantic Segmentation

Open-Vocabulary Semantic Segmentation (OVSS) enables models to recognize categories that are absent from the training set, offering a promising solution to the limitations of the closed-set paradigm in remote sensing image segmentation. Directly transferring general OVSS approaches to remote sensing scenarios remains non-trivial. The overhead imaging perspective in remote sensing introduces systematic discrepancies with natural images, which leads to misalignment in vision–language models such as CLIP, while also making it more difficult to recover fine-grained spatial details during feature upsampling. Meanwhile, the hierarchical organization and complex semantic relationships among land cover categories further challenge the representational capacity of generic text embeddings.

To address these challenges, recent studies have advanced OVSS in remote sensing along several complementary directions, forming an increasingly diverse methodological landscape.

One line of work focuses on improving feature representation and refining the foundation framework. Li et al. [118] proposed SegEarth-OV, which introduces a training-free OVSS paradigm into the remote sensing domain. To mitigate the sensitivity to low-resolution features, a universal upsampler, SimFeatUp, is designed to recover spatial information from deep features using a small number of unlabeled images. At the same time, a simple subtraction operation is applied to compensate for the global bias observed in CLIP patch tokens. Empirical results across 17 datasets indicate consistent improvements across multiple tasks, including semantic segmentation, building extraction, road detection, and flood detection. However, the effectiveness of SimFeatUp depends on the diversity and quality of the unlabeled data, and may degrade in homogeneous or noisy scenes. Moreover, the subtraction-based correction remains heuristic in nature, and its generalizability across sensors and resolutions has not yet been sufficiently validated.

Beyond feature enhancement, another direction explores the introduction of structured knowledge to improve semantic representation. The KG-OVRSeg framework [75] constructs a land cover concept knowledge graph to encode relationships such as hypernymy, synonymy, and containment. Through subgraph querying and embedding aggregation, it generates more discriminative category representations and achieves superior performance on benchmarks such as OpenEarthMap, Potsdam, and LoveDA. The effectiveness of this approach depends critically on the quality of the knowledge graph. Errors or missing relationships can be propagated through the aggregation process, while the construction, maintenance, and dynamic updating of the graph introduce non-trivial overhead in practical applications.

A related line of research investigates the role of scene context in guiding open-vocabulary segmentation. Huang et al. [77] proposed the SCORE framework, which introduces open-vocabulary learning into remote sensing instance segmentation by leveraging a multi-granularity scene context fusion mechanism. By jointly enhancing visual and textual features, the model improves instance-level prediction for unseen categories. Compared with knowledge-driven approaches such as KG-OVRSeg, this formulation places greater emphasis on global scene context as a guiding signal for instance-level prediction. Its effectiveness may be limited in scenarios with highly homogeneous semantics or extremely dense object distributions, where scene-level cues become less discriminative.

Alongside these developments, SAM-based collaborative frameworks have been explored to bridge the gap between spatial perception and semantic understanding. AerOSeg [119] integrates SAM and CLIP by computing direction-invariant image–text correlations from multiple rotated inputs and refining them through a SAM-guided Swin Transformer module. This design combines the spatial precision of SAM with the semantic generalization capability of CLIP, leading to consistent improvements on benchmarks such as iSAID, DLRSD, and OpenEarthMap. The use of multiple rotated inputs significantly increases computational overhead, limiting its applicability in large-scale or real-time scenarios. In addition, the sensitivity of the refinement process to initialization and iteration settings remains insufficiently studied. Moving toward a more data-centric paradigm, SkySense-O [120] introduces a large-scale pixel-level open-text annotation dataset (Sky-SA), constructed through a combination of GPT-4V-based description generation, model-assisted pre-annotation, and manual verification. Together with the SkySense visual foundation model, it shows consistent zero-shot performance across 14 datasets. This approach expands both data scale and task coverage, but relies on costly annotation pipelines and specific foundation models, raising concerns about scalability and long-term sustainability.

Recent advances indicate a gradual move toward more robust semantic alignment and integration strategies, where diverse mechanisms such as feature enhancement, structured knowledge, and cross-model collaboration are combined to improve generalization to unseen categories.

### 4.4. Domain Adaptation and Transfer Learning

In practical remote sensing applications, imagery is often acquired across heterogeneous sensors, geographic regions, and temporal spans. This diversity inevitably introduces pronounced domain shifts. Conventional domain adaptation techniques typically depend on a substantial amount of labeled target-domain data, which is often infeasible in time-critical scenarios such as disaster emergency response and rapid environmental monitoring. This constraint has driven increasing attention toward adaptation under extremely limited supervision, where only a few or even a single target-domain sample is available.

Despite growing efforts in this direction, existing studies remain fragmented. Current solutions are largely developed along separate technical routes, ranging from one-shot rapid adaptation to geographical prior modeling and pseudo-label optimization. These directions are rarely examined within a unified framework, which limits their collective effectiveness in practical deployment.

One line of work tackles the problem from the perspective of rapid adaptation with minimal target supervision. Tan et al. [121] introduce the Minimax One-shot AdapTation (MOAT) framework, which formulates adaptation as a minimax optimization process. The method alternates between maximizing cross-entropy to identify the most informative source-domain sample and minimizing the cross-entropy of the selected sample to align the model with the target domain. Through this adversarial procedure, MOAT enables effective adaptation using only a single unlabeled target sample and demonstrates clear advantages over conventional approaches in cross-domain settings such as urban-to-rural and rural-to-urban segmentation. This efficiency is particularly valuable in time-critical scenarios, such as disaster assessment, where rapid adaptation is essential. Its performance is closely tied to the selection of the informative sample. When the chosen target instance fails to reflect the overall distribution, the optimization process may converge to suboptimal local minima. In addition, existing evaluations are largely confined to relatively structured scenarios, leaving its generalization ability in more challenging settings—such as optical-to-SAR adaptation—insufficiently explored.

A complementary perspective focuses on the role of auxiliary prior knowledge, particularly geographic information. Verma et al. [122] propose SegDesic-Net, which embeds geographic coordinates into learnable representations and integrates them into the segmentation network. By leveraging spatial regularities—for instance, the tendency of regions with similar latitudes to share vegetation characteristics—this approach provides complementary cues for cross-domain generalization beyond purely visual features. Empirical evidence suggests that such geographic embeddings can significantly improve performance on unseen regions. The underlying assumption of geographic similarity does not always hold, especially in cross-climate zones or areas heavily influenced by human activities such as rapid urban expansion. Furthermore, the effectiveness of this strategy depends on sufficient geographic coverage in the training data; when the target region lies far outside the sampled distribution, the reliability of extrapolation remains uncertain.

Beyond explicit prior modeling, another emerging perspective focuses on improving pseudo-label quality through data distribution modeling. DiffMatch [123] and related diffusion-based semi-supervised frameworks, although originally developed for natural images, highlight the potential of generative models to capture intrinsic data distributions and stabilize pseudo-label generation. This capability is particularly relevant to remote sensing scenarios, where unreliable pseudo-labels often become a bottleneck under few-shot settings. In principle, diffusion models offer a potentially more robust mechanism for pseudo-label refinement when labeled data is extremely scarce. This line of research remains relatively underexplored in the context of remote sensing. Several key challenges are still unresolved, including adaptation to multi-spectral inputs, large-scale imagery, and high-resolution data. Moreover, the high computational cost of diffusion-based inference stands in tension with the efficiency requirements of large-scale remote sensing applications, leaving the practicality of direct transfer to be validated.

Taken together, these approaches provide complementary perspectives on few-shot domain adaptation. Nevertheless, a fundamental tension remains between their underlying assumptions and the complexity of real-world remote sensing scenarios, where domain shifts are often multi-source, cross-temporal, and highly heterogeneous. Bridging this gap requires not only better integration of these strategies, but also a clearer understanding of when reliable adaptation is possible under extremely limited supervision.

## 5. Challenges and Future Prospects

Although recent foundation models have substantially improved the representation capability of remote sensing image segmentation through architectural evolution, large-scale pre-training, and multimodal learning paradigms, current progress still reveals a fundamental tension between representation generality and remote sensing-specific adaptation.

On the one hand, foundation models aim to learn universal representations transferable across tasks and domains; on the other hand, remote sensing imagery exhibits strong modality heterogeneity, extreme scale variation, and complex spatio-temporal dependencies that remain difficult to fully capture through general-purpose pre-training alone. Consequently, many adaptation strategies discussed in Section 4 can be interpreted as downstream efforts to compensate for the residual mismatch between general foundation models and remote sensing data. At the architectural level, existing frameworks still face a trade-off between representation capability and computational efficiency. Pure Transformer architectures such as ViT suffer from severe memory bottlenecks on ultra-high-resolution imagery, whereas state space models such as Mamba, despite their linear complexity, still exhibit limitations in modeling two-dimensional spatial topology. In addition, the design of multi-directional scanning strategies still lacks sufficient theoretical support for remote sensing scenarios. Meanwhile, the issue of multimodal heterogeneity in the remote sensing field has not yet been fundamentally resolved. Sensor data from optical, SAR, multispectral, and LiDAR sources differ essentially in imaging mechanisms, noise characteristics, and semantic granularity. The strong dependence of existing multi-source fusion pre-training frameworks, such as the SkySense series, on spatio-temporal registration accuracy and the influence of cross-sensor domain shift on feature alignment stability both lack sufficient theoretical analysis and ablation study support in current research [84]. In addition, the feature distribution gap between general segmentation foundation models such as SAM and the remote sensing field—which encompasses overhead perspective bias, multi-spectral band semantics, and ground object density distribution—still persists as a residual gap across various adaptation methods, rather than being fundamentally addressed during the pre-training stage. This inherent limitation is reflected across multiple technical routes, including parameter-efficient fine-tuning, prompt engineering, and domain adaptation.

Beyond architectural and adaptation limitations, the current data and evaluation ecosystem remains insufficient to fully support the development of remote sensing foundation models. Existing remote sensing image segmentation benchmarks exhibit significant limitations in geographical coverage, sensor type, spatial resolution gradient, and annotation granularity, which constrain the objective assessment of a method’s true generalization ability. The scarcity of high-quality pixel-level language–vision annotation data constitutes a fundamental bottleneck restricting the training quality of Multimodal Large Language Models (MLLMs). Moreover, the substantial discrepancies among current datasets in category definition (ranging from 2 to 274 classes) and annotation paradigm (semantic, instance, and panoptic segmentation) undermine the reliability of cross-dataset performance comparisons. These inconsistencies are further amplified by annotation uncertainty, including ambiguous object boundaries, mixed pixels, weakly defined land-cover transitions, and differences in annotator criteria across datasets. More importantly, current evaluation systems provide only limited coverage of key practical attributes such as uncertainty quantification, adversarial robustness, long-tail category handling, open-vocabulary validity, and edge device deployability. This gap reflects a fundamental mismatch between existing evaluation practices and the practical demands of remote sensing image segmentation in high-timeliness scenarios such as disaster emergency response and land monitoring. In particular, open-vocabulary and vision–language segmentation methods are still mainly validated on limited benchmarks, and their robustness to unseen categories, sensor shifts, geographic transfer, and operational deployment remains insufficiently tested. The theoretical boundaries of few-shot domain adaptation also remain unclear, particularly regarding target-domain representativeness, geographical prior transferability, and generative pseudo-label optimization under remote sensing conditions.

These gaps suggest that future research should focus on several more concrete and testable directions. A first direction is sensor-aware remote-sensing-native pre-training. Instead of directly extending natural-image pre-training objectives to remote sensing data [124], future studies need to examine how SAR structure, multispectral characteristics, spatial resolution differences, and temporal dynamics can be jointly modeled within a unified pre-training objective while reducing the dependence on precisely registered multi-source data. Potential technical routes include sensor-aware masking, physics-informed spectral–spatial reconstruction, cross-sensor contrastive learning, and pre-training tasks that explicitly encode spatial scale and temporal change.

A second direction is the construction of generalization-oriented and uncertainty-aware benchmarks. Future benchmarks should evaluate whether a model remains reliable under cross-region, cross-sensor, cross-resolution, and cross-season transfer, rather than only reporting performance on fixed train–test splits. Such evaluation protocols should explicitly consider annotation uncertainty, boundary ambiguity, label inconsistency, long-tail categories, and calibration quality. For open-vocabulary segmentation, evaluation should also move beyond category-level recognition accuracy and examine robustness to unseen land-cover concepts, hierarchical semantic labels, text–image misalignment, and sensor-dependent visual ambiguity.

A third direction is efficient and deployable model design for ultra-high-resolution imagery. Rather than treating deployment as a post-processing issue, future work should consider memory cost, latency, and energy consumption during model design. Mamba-based modeling, window or linear attention, low-rank adaptation, model pruning, quantization, and tile-aware inference may provide practical routes for reducing computational cost while preserving boundary quality, small-object recognition, and cross-domain robustness. This issue is particularly important for edge devices, onboard processing, and near-real-time applications.

Finally, open-world geospatial foundation models need to be evaluated in operational scenarios, not only in static benchmark settings. Although integrating open-vocabulary understanding, multimodal reasoning, geographic priors, and SAM-based interactive segmentation may support more flexible geospatial interpretation, their reliability still needs to be tested in disaster response, land monitoring, urban change analysis, and multi-temporal environmental observation. Key open questions include how to detect model failure under sensor shift, represent uncertainty in human-in-the-loop segmentation, update models with limited new annotations, and evaluate robustness when label spaces, imaging conditions, and application requirements change over time.

## 6. Conclusions

This paper presents a review of foundation model architectures and adaptation strategies for remote sensing image segmentation. From the perspective of dataset evolution, we analyze how the increasing scale, modality diversity, semantic openness, and temporal complexity of remote sensing data have driven the transition from conventional task-specific supervised models toward foundation-model paradigms. We further summarize major architectural and pre-training paradigms, including Transformer-based models, hybrid CNN–Transformer frameworks, state space models, prompt-driven segmentation models, self-supervised learning, and multimodal vision–language pre-training. In addition, recent advances in adaptation strategies, such as parameter-efficient fine-tuning, prompt engineering, few-shot and zero-shot learning, open-vocabulary segmentation, and domain adaptation, are reviewed and analyzed under heterogeneous remote sensing conditions.

Remote sensing image segmentation is evolving from closed-set supervised learning toward open-world geospatial foundation models with stronger representation, reasoning, and generalization capabilities. Despite substantial progress, several key challenges remain unresolved, including the domain gap between natural-image pre-training and remote sensing imagery, the trade-off between modeling capability and computational efficiency, multimodal and spatio-temporal representation learning, annotation uncertainty and label inconsistency, and the lack of generalization-oriented evaluation systems. Future research should focus on sensor-aware remote-sensing-native pre-training, uncertainty-aware and cross-domain benchmark construction, efficient deployable architectures for ultra-high-resolution imagery, and operational validation of open-world geospatial foundation models. It is hoped that this review can provide useful references for future research and promote the continued development of intelligent Earth observation systems.

## Figures and Tables

**Table 1:** Comparison of representative foundation-model paradigms for remote sensing image segmentation, with their roles in the segmentation pipeline.

Model	Role in Segmentation Pipeline	Technical Type	Core Mechanism	Pre-Training Strategy
ViT [3]	Visual backbone/encoder	Pure Transformer	Global self-attention over image patches	Supervised or self-supervised large-scale pre-training
Swin Transformer [7]	Hierarchical visual backbone	Hierarchical Transformer	Shifted window self-attention for local–global representation	Supervised or self-supervised pre-training
CNN-Transformer	Local–global feature backbone	Hybrid CNN + Transformer	CNN extracts local features while Transformer models global context	Supervised or self-supervised pre-training
Mamba [8]	Long-range dependency modeling	State Space Model (SSM)	Selective state-space modeling for long-range dependency	Self-supervised or supervised pre-training
SAM [4]	Promptable mask generation	ViT Encoder + Mask Decoder	Prompt-guided segmentation via mask prediction	Large-scale supervised pre-training on SA-1B
DINO [9]	Self-supervised representation learning	ViT-based Transformer	Teacher-student self-distillation for representation learning	Self-supervised self-distillation pre-training
MAE [10]	Masked image modeling pre-training	ViT-based Transformer	Masked image modeling using asymmetric encoder-decoder	Self-supervised masked reconstruction pre-training
CLIP [11]	Vision–language alignment	Vision Transformer + Text Encoder	Cross-modal contrastive learning between images and text	Large-scale image–text pre-training

Abbreviations: ViT, Vision Transformer; CNN, Convolutional Neural Network; SSM, State Space Model; SAM, Segment Anything Model; DINO, Self-Distillation with No Labels; MAE, Masked Autoencoder; CLIP, Contrastive Language–Image Pre-Training; SA-1B, Segment Anything 1 Billion.

**Table 2:** Representative, non-exhaustive summary of remote sensing datasets for semantic segmentation.

Category	Dataset	Year	Sensor/Source	Resolution	Size	Region/Coverage
Urban/Building & Infrastructure	ISPRS Vaihingen [14]	2012	Aerial imagery + DSM	9 cm GSD	33 image patches	Germany
	ISPRS Potsdam [14]	2012	Aerial imagery + DSM	5 cm	38 images (6000 × 6000 px)	Germany
	Massachusetts Roads [15]	2013	Aerial imagery	1 m	1171 images (1500 × 1500 px)	USA
	Massachusetts Buildings [15]	2013	Aerial imagery	1 m	151 images (1500 × 1500 px)	USA
Urban/Building & Infrastructure	Zurich Summer [25]	2015	QuickBird pansharpened imagery	0.62 m	20 images (1000 × 1150 px)	Switzerland
	SpaceNet (initial release) [16]	2016	WorldView-2 satellite	50 cm	200 m tiles; 382,534 bldg. footprints	Rio de Janeiro, Brazil
	Inria Aerial [26]	2017	Aerial imagery	30 cm	360 images (1500 × 1500 px)	USA & Austria
	DSTL [27]	2017	WorldView-3 satellite	0.31 m, 1.24 m, 7.5 m	57 images (3300 × 3300 px)	Not explicitly specified
	WHU Building [28]	2018	Aerial & satellite imagery	0.3 m, 2.7 m	8189/17,388 tiles (512 × 512 px)	New Zealand/ East Asia
	DeepGlobe (road/building tracks) [17]	2018	Satellite imagery	0.5 m/ 0.31 m–1.24 m	8570 road images; 24,586 bldg. scenes	Multi-country
	SkyScapes [29]	2019	Aerial imagery	13 cm	16 images (5616 × 3744 px)	Germany
	SemCity Toulouse [30]	2020	Pan-sharpened multispectral satellite imagery	50 cm	16 tiles (3504 × 3452 px)	Toulouse, France
Instance Segmentation	iSAID [18]	2019	Multi-source aerial & satellite imagery (Google Earth, etc.)	0.3–1 m	2806 images (800–13,000 px)	Multiple regions
Land-Cover/ Land-Use	DeepGlobe (land-cover track) [17]	2018	Satellite imagery	0.5 m	1146 images (2448 × 2448 px)	Multi-region
	GID [31]	2018	GF-2 satellite	4 m	150 images (6800 × 7200 px)	China
	LandCover.ai [32]	2021	Aerial imagery	0.25 m, 0.5 m	41 images (4200–9500 px)	Poland
	LoveDA [33]	2021	Google Earth satellite imagery	0.3 m	5987 images (1024 × 1024 px)	China
	DFC2022 [34]	2022	Aerial imagery (MiniFrance RGB)	0.5 m	VHR tiles (∼2000 × 2000 px)	France
	OpenEarthMap [35]	2022	Aerial & satellite imagery	0.25–0.5 m	5000 images (1024 × 1024 px)	97 regions/ 44 countries
	Five-Billion-Pixels [20]	2023	GF-2 satellite	4 m	150 images (6800 × 7200 px)	China
	FUSU [36]	2024	Google Earth + Sentinel multi-source	0.2–0.5 m	62,752 patches (512 × 512 px)	China
	OpenEarthMap-SAR [37]	2025	SAR imagery	0.15–0.5 m	5033 images (1024 × 1024 px)	Japan, France, USA
Disaster/ Environmental Monitoring	WorldFloods [21]	2023	Sentinel-2	10 m	∼180,000 patches (256 × 256 px)	Global
	Kuro Siwo [38]	2024	Multi-temporal Sentinel-1 SAR	10 m	67,490 time-series (224 × 224 px)	Global
UAV Datasets	UAVID(v2020) [19]	2020	UAV imagery	∼10 cm	300 images (3840/ 4096 × 2160 px)	Urban scenes
Multimodal/ Vision-Language/ Foundation	RefSegRS [23]	2024	Aerial imagery + language	0.13 m	4420 triplets (512 × 512 px)	Urban scenes
	EarthVQA [39]	2024	Satellite imagery (WorldView-3)	0.3 m	6000 images (512 × 512 px)	China
	NWPU-Refer [24]	2025	Satellite imagery + language	0.12–0.5 m	15,003 images (1024–2048 px)	30+ countries
	EarthReason [40]	2025	Satellite imagery	0.5–153 m	5434 images (1232–7617 px)	Global
	GeoSeg-1M/Ge oSeg-Bench [22]	2025	Multi-source satellite + instructions	0.05–153 m	590,000 images; 1.1 M triplets (512 × 512 px)	Global

**Table 3:** Evolutionary comparison of the DINO series from DINOv1 to DINOv3.

Comparison Dimension	DINOv1 (2021) [9]	DINOv2 (2023) [62]	DINOv3 (2025) [65]
Core Idea	Self-distillation with multi-view consistency	Scalable self-supervision with hybrid objectives	Structure-preserving learning via feature correlation constraints
Training Objective	Global self-distillation (DINO loss)	DINO + iBOT + KoLeo regularization	DINO + iBOT + KoLeo + Gram Anchoring
Data Scale	ImageNet-1K (∼1 M)	LVD-142M (142 M curated)	LVD-1689M (1.69 B), SAT-493M
Model Scale	ViT-S/B (5.6–86 M)	ViT-g (22 M–1.1 B)	ViT-7B (22 M–6.7 B)
Position Encoding	Learnable absolute PE	Learnable PE + resolution adaptation	Rotary positional embedding (RoPE)
Architecture Details	Standard ViT, single head	Register tokens, MemEffAttention	Registers, RoPE jitter, RMSNorm
Training Complexity	Medium	High	High but more stable optimization
Representation Level	Image-level	Image + patch-level	Multi-granularity with structure constraints
Performance Characteristics	Emergent object-centric attention	Strong classification and dense prediction	Mitigates dense feature collapse; better zero-shot

Note: DINOv3 is included as a recently released technical report/open model rather than a peer-reviewed conference or journal publication.

**Table 4:** Comparison of LoRA- and adapter-based PEFT strategies for remote sensing image segmentation.

PEFT Strategy	Representative Methods	Adapted Components	Parameter/Cost Profile	Benchmark Evidence	Main Strengths and Limitations
LoRA	LoRA [64]; Conv-LoRA [87]; PriorSAM-DBNet [89]	Low-rank updates in Transformer/SAM attention layers; variants add convolutional priors or lightweight task branches	Few trainable parameters; standard LoRA is mergeable, while structural variants may add inference cost; PriorSAM-DBNet fine-tunes only 0.91 M parameters	Remote sensing segmentation-related tasks, including building extraction and video segmentation	Efficient and deployment-friendly, but rank/layer choices and added task-specific modules remain empirical; cross-study results are not directly comparable
Adapter	SAM-Adapter [93]; MSA-SAM [97]; CWSAM [96]	Lightweight adapters in the SAM image encoder; variants add task-specific decoders or feature-enhancement modules	Fine-tunes only adapter and lightweight task-specific modules; frozen SAM backbone; additional modules remain active during inference	Mainly SAM adaptation for optical and SAR remote sensing semantic segmentation	Flexible for domain adaptation, but relies on task-specific module design and usually incurs higher inference complexity than mergeable LoRA

**Table 5:** Comparison of major SAM usage and adaptation modes for remote sensing image segmentation.

SAM Usage/ Adaptation Mode	Representative Methods	Core Mechanism	Prompt/Supervision Requirement	Remote-Sensing Target	Main Strengths and Limitations
Interactive zero-shot use of original SAM	SAM [4]	Uses the original SAM encoder, prompt encoder, and mask decoder for prompt-conditioned mask prediction	Requires manual points, boxes, or masks; no task-specific fine-tuning	Interactive object-level mask generation in remote sensing images	Flexible for interactive annotation, but limited by manual prompting, category-agnostic masks, weak semantic discrimination, high feature density, and domain gaps
Automated prompt generation	RSPrompter [98]; RSPS-SAM [103]; RSAM-Seg [99]	Generates category-specific or visual-feature-guided prompts; may embed prompt generation into the segmentation pipeline	Reduces manual prompts; usually requires task-specific training labels	Large-scale automated remote sensing segmentation	Improves automation, but prompt quality is sensitive to proposal quality, early feature ambiguity, dense objects, and weak boundaries
Parameter-efficient SAM adaptation	SAM-Adapter [93]; MSA-SAM [97]; CWSAM [96]	Inserts lightweight adapters or task-specific modules into the SAM encoder/decoder to inject domain, scale, or modality priors	Requires downstream training data; avoids full fine-tuning of SAM	Optical and SAR remote sensing semantic segmentation	Better domain adaptation and boundary/scale modeling, but adds architectural complexity and may reduce compatibility with the original prompt-driven interface
Text-guided SAM pipelines	RS2-SAM2 [104]	Adapts SAM/SAM2 with visual-textual joint encoding, multimodal prompt tokens, and hierarchical cross-modal fusion	Requires text prompts or referring expressions	Referring remote sensing image segmentation	Improves semantic controllability, but remains sensitive to text–image alignment quality and has uncertain transferability to general semantic segmentation

Note: The modes are not strictly mutually exclusive. Some methods combine automatic prompting, adapter-based tuning, and language-guided grounding; the table highlights their dominant role in adapting SAM to remote sensing segmentation.

## Data Availability

No new data were created or analyzed in this study. Data sharing is not applicable to this article.
